# Small Fiber Neuropathy After SARS-CoV-2 Infection and Vaccination: A Case-Based Comparison

**DOI:** 10.7759/cureus.43600

**Published:** 2023-08-16

**Authors:** Beth Schwartz, Shyam Moudgil, Frank Pollina, Ashish Bhargava

**Affiliations:** 1 Internal Medicine, Ascension St. John Hospital, Detroit, USA; 2 Neurology, Ascension St. John Hospital, Detroit, USA; 3 Physical Medicine and Rehabilitation, Ascension St. John Hospital, Detroit, USA

**Keywords:** sars-cov-2, small fiber neuropathy, covid-19, covid-19 and neurology, covid-19 mrna vaccine, long covid symptoms

## Abstract

COVID-19-associated neuropathies, whether post-infection or post-vaccination, have not been fully described. A variety of theories exist to explain these phenomena, many of them centering on immune dysregulation. We aim to contribute to the discussion on the similarities and differences behind the two conditions and to bolster the call for further research to be done in this area. We will discuss two different case presentations, one patient experiencing a post-COVID-19 infection neuropathy and the other experiencing a post-COVID-19 vaccination neuropathy.

## Introduction

More than 20 million people in the United States suffer from peripheral neuropathy [1], and up to 46% of these cases are deemed idiopathic [2]. At the time this manuscript was written, over 100 million people in the United States have had a reported COVID-19 infection [3]. Data regarding the prevalence of post-COVID-19 conditions are still developing; however, one of the largest studies to date identified 6.2% of people previously infected with COVID-19 to have post-COVID-19 symptoms [4]. This study did not include those infected with the Omicron variant, and therefore, further research is necessary to identify the incidence and prevalence of post-COVID-19 symptoms and the potential treatments for this condition [4]. Moreover, at the time this manuscript was written, over 670 million doses of COVID-19 vaccines have been administered [5]. Post-vaccination neuropathy appears to be exceedingly rare, with prior estimates ranging from 0.01% to 0.13% [6]. Here, we highlight two different cases, one of post-infection and one of post-vaccination neuropathy with the hope to compare and contrast the possible mechanisms behind these conditions.

## Case presentation

Case #1

A 44-year-old female with no medical history presented with left-sided facial numbness two days after receiving a messenger ribonucleic acid (mRNA) COVID-19 booster dose. The numbness was associated with decreased sensation in the mouth and loss of taste on the left side of the tongue. She endorsed severe burning from the lower border of the maxilla to the mandible, lips, and chin. Steroids and gabapentin provided no relief. She also experienced numbness and severe burning in the right leg after her first COVID-19 vaccine 14 months prior, which has been persistent. Laboratory results, including complete blood count, complete metabolic panel, vitamin B12 level, and erythrocyte sedimentation rate, were within normal limits (Table 1). Electromyography (EMG) was negative. Magnetic resonance imaging (MRI) of the brain was negative for demyelination. She underwent nerve stimulator placement with improvement in pain. She uses carbamazepine for her symptoms, which provides minimal relief.

**Table 1:** Laboratory values for the patient in case #1 CO2 = carbon dioxide; GFR = glomerular filtration rate; MCHC = mean corpuscular hemoglobin concentration; RDW = red cell distribution width; WBC = white blood cells.

Laboratory test	Result	Reference range
Urea nitrogen	12	8-20 mg/dL
Creatinine	0.81	0.70-1.20 mg/dL
Urea nitrogen/creatinine ratio	14.8	12.0-20.0
GFR, calculated	90	60-130
Sodium	138	135-145 mmol/L
Potassium	4.5	3.5-5.2 mmol/L
Chloride	105	98-109 mmol/L
Anion gap	8	4-14 mmol/L
CO2 content	25	23-34 mmol/L
Calcium	9.0	8.4-10.5 mg/dL
Glucose - fasting	90	70-99 mg/dL
Bilirubin total	<0.2	0.0-1.5 mg/dL
Alkaline phosphatase	66	20-120 IU/L
Alanine aminotransferase	25	0-40 U/L
Aspartate aminotransferase	26	0-35 IU/L
Total protein	6.8	6.2-8.1 g/dL
Albumin	4.0	3.5-5.0 g/dL
Globulin	2.8	1.8-3.6 g/dL
Hemoglobin	13.6	12.0-16.0 g/dL
Hematocrit	41.4	36.0-46.0%
Red blood cell count	4.46	4.00-5.40 M/uL
Mean corpuscular volume	92.8	80-102.0 f
Mean corpuscular hemoglobin	30.5	26.0-34.0 pg
MCHC	32.9	31.0-37.0 g/dL
RDW	12.7	11.0-15.5%
Platelet count	270	150-400 K/uL
WBC	5.83	4.00-11.00 K/uL
Erythrocyte sedimentation rate	8	2-37 mm/hr
Vitamin B12	371	232-1245 pg/mL

Case #2

A 54-year-old female with Hashimoto thyroiditis presented following two COVID-19 infections, the first in December 2020 and the second in September 2022. She tested positive for COVID-19 in December 2020 and experienced mild upper respiratory symptoms without hypoxia. She developed sensory loss in the upper extremities. Upon following up three months later, her symptoms had improved. In October 2021, she noticed worsening burning sensations in her shoulders and thighs. As she was unvaccinated, anti-spike immunoglobulin G (IgG) was collected and found to be elevated. In September 2022, she developed upper respiratory infection symptoms and tested positive for COVID-19 again. She had persistently elevated anti-spike IgG eight months after reinfection. She developed worsening burning and numbness in her lower extremities. The complete metabolic panel, complete blood count, sedimentation rate, hemoglobin A1c, vitamin B12, and vitamin D were within normal limits (Table 2). The patient was evaluated with an EMG as well as an MRI of the brain and lumbar spine to evaluate for structural causes of her symptoms, which were negative. She denied improvement with amitriptyline, gabapentin, pregabalin, or capsaicin.

**Table 2:** Laboratory values for the patient in case #2 CO2 = carbon dioxide; GFR = glomerular filtration rate; MCHC = mean corpuscular hemoglobin concentration; RDW = red cell distribution width; WBC = white blood cells.

Laboratory test	Result	Reference range
COVID-19 anti-spike IgG	119.5	<25 IU/mL
Hemoglobin A1c	5.3	4.0-6.0%
Urea nitrogen	20	8-20 mg/dL
Creatinine	0.95	0.70-1.20 mg/dL
GFR, calculated	72	60-130 mL/min/1.73 m2
Sodium	139	135-145 mmol/L
Potassium	4.5	3.5-5.2 mmol/L
Chloride	101	98-109 mmol/L
Anion gap	10	4-14 mmol/L
CO2 content	28	23-34 mmol/L
Glucose - random	94	70-200 mg/dL
Calcium	9.5	8.4-10.5 mg/dL
Bilirubin, total	0.9	0.0-1.5 mg/dL
Alkaline phosphatase	45	20-130 IU/L
Alanine aminotransferase	25	0-40 U/L
Aspartate aminotransferase	30	0-35 IU/L
Total protein	6.5	6.2-8.1 g/dL
Albumin	4.5	3.5-5.0 g/dL
Globulin	2.0	1.8-3.6 g/dL
Hemoglobin	13.3	12.0-16.0 g/dL
Hematocrit	41.3	36.0-46.0%
Red blood cell count	43.5	4.00-5.40 M/uL
Mean corpuscular volume	94.9	80.0-102.0 fL
Mean corpuscular hemoglobin	30.6	26.0-34.0 pg
MCHC	32.2	31.0-37.0 g/dL
RDW	11.9	11.0-15.5%
Platelet count	233	150-400 K/uL
WBC	5.43	4.00-11.00
Erythrocyte sedimentation rate	8	2-39 mm/hr
Ferritin	78	8-120 NG/mL
Vitamin B12	471	232-1245 pg/mL
Vitamin D,1,25-dihydroxy	37.7	24.8-81.5 pg/mL

## Discussion

These patients may have idiopathic small fiber neuropathy, characterized by damage to peripheral nerves. Symptoms of small fiber neuropathy are varied, including allodynia and hyperesthesia [7]. EMGs are generally focused on larger nerves and may be negative in small fiber neuropathy [7]. Skin biopsy is the most evidence-based technique to diagnose small fiber neuropathy [7]. The cases presented here are limited by the lack of skin biopsy; however, the clinical picture may be consistent with small fiber neuropathy. The sensory distribution described in case #1 can be consistent with trigeminal neuralgia; however, the trigeminal nerve also contains small fibers and thus can be affected by small fiber neuropathy [8]. Though small fiber neuropathy is associated with stocking distribution of neuropathy, non-length dependent cases of proximal polyneuropathies and those involving the face also exist and are rare manifestations. Small fiber neuropathy is associated with human immunodeficiency virus (HIV), hepatitis C, Guillain-Barre syndrome, systemic lupus erythematosus, and paraneoplastic syndromes. Proposed mechanisms of small fiber neuropathy include autoantibodies to neuronal proteins, inflammatory cytokines in the skin, and vasculitis [7].

In post-COVID-19 infection-associated small fiber neuropathy, several mechanisms have been proposed. Autoimmune findings have been associated with the presence of the humoral response to COVID-19, such as the formation of autoantibodies against interleukin-2 and interferon-gamma, among others [9]. The patient discussed in case #2 had positive anti-spike IgG levels; further research examining if this correlates to the development of an autoimmune neuropathy may prove useful. Small fiber neuropathy appears to be a common finding in patients with post-COVID-19 symptoms. One study by Oaklander et al. (2022) analyzed 17 patients with post-COVID-19 symptoms and found small fiber neuropathy to be the most common type of neuropathy experienced [10]. Although limited by sample size, this study discussed how small fibers themselves are at risk for damage, given their lack of myelin protection, high metabolic needs, and limited ability to reconstruct axons.

In discussing COVID-19 vaccines, we highlight mRNA vaccines, as this is the vaccine type our patient received. One proposed mechanism of post-vaccine neuropathy is through molecular mimicry. COVID-19 infection can create autoantibodies, and it was previously hypothesized that COVID-19 vaccination may also create autoantibodies. This theory has been questioned due to the difference in viral and human peripheral nerve protein structure; however, no research has definitively refuted this claim [11]. Rates of post-COVID-19 neuropathy are exceedingly low; previous cases have detailed it to be on the order of roughly 0.01-0.13% [6]. Additional data regarding the management of post-vaccination neuropathy would be helpful for the patients affected.

## Conclusions

Of the mechanisms discussed, immune dysregulation appears to be the commonality. Further research into the exact mechanisms of these neuropathies is warranted. Both post-infectious and post-vaccination neuropathies have been hypothesized to be a result of an autoimmune phenomenon. This raises additional questions as to whether those with an underlying autoimmune condition have a predilection to an autoimmune response to COVID-19 infection itself or its vaccine. Larger studies stratifying the risk based on the presence of autoimmune disease and the development of either type of neuropathy may prove illuminating. Additional data regarding the exact prevalence of post-vaccination neuropathy would also help practitioners better address questions from patients surrounding vaccination.
